# *Drosophila* OTK Is a Glycosaminoglycan-Binding Protein with High Conformational Flexibility

**DOI:** 10.1016/j.str.2020.02.008

**Published:** 2020-05-05

**Authors:** Daniel Rozbesky, Jim Monistrol, Vitul Jain, James Hillier, Sergi Padilla-Parra, E. Yvonne Jones

**Affiliations:** 1 Division of Structural Biology, Wellcome Centre for Human Genetics, University of Oxford, Oxford OX3 7BN, UK; 2 Cellular imaging, Wellcome Centre for Human Genetics, University of Oxford, Oxford OX3 7BN, UK; 3 Department of Infectious Diseases, King's College London, Faculty of Life Sciences & Medicine, London SE1 9RT, UK; 4 Randall Centre for Cell and Molecular Biology, King's College London, London SE1 1UL, UK

**Keywords:** OTK, Off-track, plexin, Wnt, GAG, glycosaminoglycans, Ig-like domain, signaling, PlexA, Sema1a

## Abstract

The transmembrane protein OTK plays an essential role in plexin and Wnt signaling during *Drosophila* development. We have determined a crystal structure of the last three domains of the OTK ectodomain and found that OTK shows high conformational flexibility resulting from mobility at the interdomain interfaces. We failed to detect direct binding between *Drosophila* Plexin A (PlexA) and OTK, which was suggested previously. We found that, instead of PlexA, OTK directly binds semaphorin 1a. Our binding analyses further revealed that glycosaminoglycans, heparin and heparan sulfate, are ligands for OTK and thus may play a role in the Sema1a-PlexA axon guidance system.

## Highlights

• *Drosophila* OTK exhibits extensive interdomain flexibility
• OTK interacts with glycosaminoglycans
• OTK directly interacts with Sema1a but not PlexA

## Graphical Abstract

**Figure:**
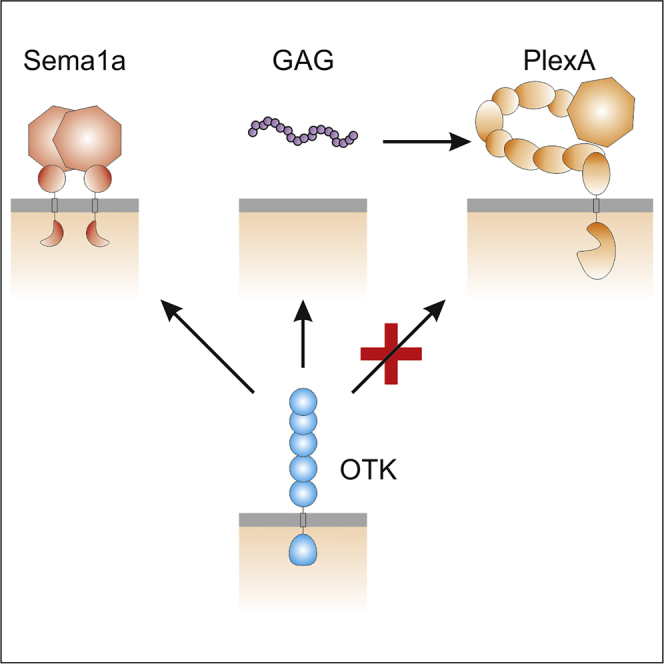

## Teaser

*Drosophila* OTK is a single-spanning transmembrane protein that plays essential roles in development and reproduction. Here, Rozbesky et al. report on the crystal structure of the last three domains of the OTK ectodomain. Binding studies highlight that OTK directly binds glycosaminoglycans and an axon guidance molecule, semaphorin 1a.

## Introduction

*Drosophila* off-track or OTK is a transmembrane protein that plays important roles in development and reproduction. OTK, previously called Dtrk, was initially identified as a neural cell adhesion molecule (Pulido et al., 1992). Nine years later, Dtrk was renamed to off-track due to disruptions of axon tract morphology observed in mutant embryos (Winberg et al., 2001). During *Drosophila* embryogenesis, OTK has been reported to show a highly dynamic expression pattern in a variety of cells, including the developing central nervous system (Pulido et al., 1992), developing photoreceptor neurons (Cafferty et al., 2004), the visceral mesoderm, the gut, the Malpighian tubules, the leg imaginal discs, and male and female genital discs (Linnemannstons et al., 2014). Sequence analysis suggests that OTK is a single-pass transmembrane protein with five extracellular immunoglobulin (Ig)-like domains, which show homology with neural cell adhesion molecules (Pulido et al., 1992). The intracellular domain shows homology with receptor tyrosine kinases; however, the kinase domain is probably not active since conserved residues implicated in autophosphorylation are altered in OTK. *Drosophila* OTK has also been described as an ortholog of the vertebrate protein tyrosine kinase 7 (PTK7), which was identified as a Wnt co-receptor required for control of planar cell polarity (Lu et al., 2004). Recent studies identified an OTK paralog, OTK2, that is most likely a result of gene duplication and is co-expressed with OTK throughout embryonic and larval development. OTK2 comprises only three extracellular Ig-like domains and a short cytoplasmic domain (Linnemannstons et al., 2014).

Functionally, OTK has been reported to bind *Drosophila* Plexin A (PlexA), a receptor for the axon guidance molecule semaphorin 1a (Sema1a). Furthermore, *in vivo*, OTK mutants showed guidance defects of certain embryonic motor axons. This phenotype resembles those of loss-of-function mutations of either PlexA or Sema1a, suggesting that OTK is involved in semaphorin-plexin signaling during axon guidance (Winberg et al., 2001). OTK has also been demonstrated to be required for lamina-specific targeting of photoreceptor axons in the developing eye, a function that is probably independent of Sema1a signaling (Cafferty et al., 2004). Previous work on Wnt signaling showed that a loss-of-function mutation of *otk* is embryonic lethal and affected embryonic cuticular patterning in a similar manner to vertebrate PTK7. Furthermore, the authors suggested that OTK interacts with Wnt4 and activates non-canonical Wnt signaling (Peradziryi et al., 2011). However, these findings are in contrast with a subsequent report, which showed that flies lacking both OTK and OTK2 are viable, although males are sterile due to defective morphogenesis of the ejaculatory duct (Linnemannstons et al., 2014). Instead of Wnt4, OTK and its paralog, OTK2, have been shown to function as co-receptors for Wnt2. Most recently, OTK and OTK2 have also been suggested to interact with *Drosophila* Ror, a nervous system-specific co-receptor for Wnt ligands (Ripp et al., 2018).

Here, we determined a crystal structure of the last three domains of *Drosophila* OTK. We found that the OTK ectodomain can adopt multiple conformations due to interdomain flexibility. We further discovered that OTK interacts with glycosaminoglycans, which could explain the ability of OTK to form complexes with numerous structurally divergent proteins.

## Results

### OTK_3-5_ Exhibits Extensive Interdomain Flexibility

**Figure 1:**
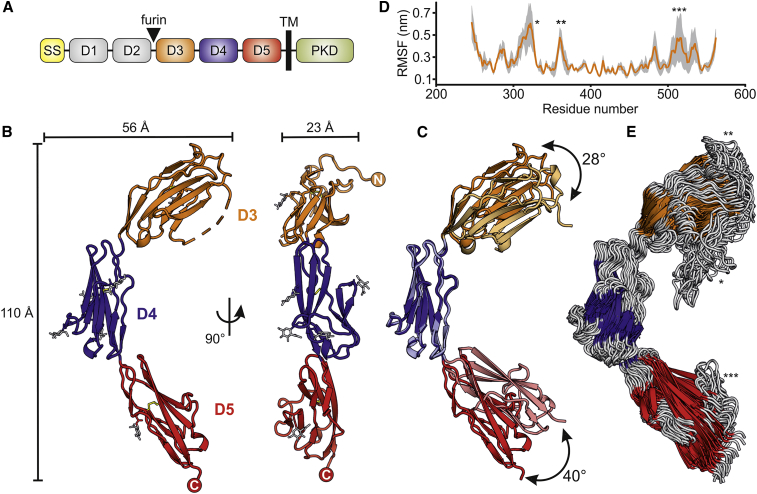
Crystal Structure of *Drosophila* OTK_3-5_ (A) Schematic domain organization of *Drosophila* OTK (SS, signal sequence; TM, transmembrane region; PKD, protein tyrosine kinase domain). (B) Ribbon representation of OTK_3-5_; N-glycans and disulfide bonds are shown in stick representation (gray and yellow, respectively), the βC′-βD loop in D3 (dashed line) was not modeled because of fragmentary electron density. (C) Superposition of OTK_3-5_ chain A (dark shades) and chain B (light shades) via the D4 domain revealed the interdomain flexibility between the Ig domains. (D) Molecular dynamics simulations of OTK_3-5_. Average root-mean-square fluctuations (RMSF) of Cα atoms (orange) were calculated from three independent simulations; the standard deviation is shown in gray. A higher level of fluctuations was observed for three loops (shown by asterisks), particularly βC′-βD and βF-βG in D3 and βC′-βD in D5. (E) Superposition of 25 Cα-traced conformers of OTK_3-5_ extracted at 500-ps intervals in the molecular dynamics simulations showed the interdomain flexibility between the Ig domains.

The entire ectodomain of *Drosophila* OTK comprises five Ig domains, designated D1-D5 (Figure 1A). We crystallized the OTK ectodomain and determined the crystal structure of the last three D3-D5 domains (OTK_3-5_) to 1.97 Å resolution (Table 1). The overall architecture of OTK_3-5_ is arranged in an extended conformation with substantial curvature resembling a boomerang shape (Figure 1B). OTK D3-D5 domains adopt the I-set fold of the Ig-like domain superfamily which has been found in many cell adhesion molecules, protein tyrosine kinase receptors, and signaling molecules (Harpaz and Chothia, 1994). These β sandwich folds are formed by two β sheets made up of strands ABBʹDE and AʹCFG, which are connected by a disulfide bond between strands B and F (Figure S1). The D4 and D5 domains are closely related and structurally most similar to the fifth Ig domain of the axon guidance molecule, human Robo1, with a root-mean-square deviation (RMSD) of 1.58 and 1.25 Å over 84 and 89 matched Cα positions, respectively. The D3 domain is most similar to the Ig-cell adhesion molecule domain (IgCAM3) of human MLCK1 with an RMSD of 1.71 Å over 89 matched Cα positions. The asymmetric unit contains two chains, and structural superposition of these chains revealed that D3 and D5 can re-orientate relative to the D4 domain by 28° and 40° about hinge points located in the D3-D4 and D4-D5 linkers, respectively, giving clear evidence for interdomain flexibility (Figure 1C). The interdomain flexibility is presumably allowed by the small interdomain interfaces and a lack of strong interactions between the individual domains. The interdomain interface between D3 and D4 buries only about 376 Å^2^ and involves hydrogen bonds between Q376-E268, Q402-E268, and T404-V373. The D4-D5 interface is even smaller, with a total buried surface area of 328 Å^2^ (chain A). The flexibility is also apparent in molecular dynamics simulations. Ten-nanosecond simulations on OTK_3-5_ showed substantial motions about the hinge points between the domains. The interdomain movement around the D4-D5 hinge point varied by 63° and was larger than the movement around D3-D4, which varied by 44° (Figures 1D and 1E). The OTK_1-5_ ectodomain contains nine potential sites for N-linked glycosylation, eight of which are located in OTK_3-5_. N-linked glycans were clearly visible and unambiguously fitted into the electron density at Asn336, 417, 429, 444, 457, and 524. Among OTK_3-5_ domains, D4 shows the highest level of N-linked glycosylation with four N-linked glycans.

**Table 1:** Data Collection and Refinement Statistics

	OTK_3-5_
**Data Collection**	

Space group	C 2 2 2_1_
Cell dimensions	
a, b, c (Å)	81.0, 189.9, 131.9
α, β, γ (°)	90, 90, 90
Resolution (Å)	46.65–1.97 (2.04–1.97)
Unique reflections	71,214 (6,384)
Multiplicity	5.9 (3.5)
Completeness (%)	97.29 (85.14)
I/σ(I)	11.0 (1.2)
Wilson B factor (Å^2^)	32.20
R_meas_ (%)	8.3 (83.5)
CC½	1.0 (0.5)

**Refinement**	

Reflections used in refinement	70,202 (6,064)
R_work_/R_free_ (%)	20.53/23.61
No. of atoms	5,294
Protein	4,660
Ligands	203
Solvent	431
B factor (Å^2^)	
Protein	49.26
Ligand	71.71
Solvent	49.63
Root-mean-square deviation	
Bond lengths (Å)	0.010
Bond angles (°)	1.32
Ramachandran plot (%)	
Favored	97.82
Allowed	2.18
Outliers	0

Highest-resolution shell is shown in parentheses.

### OTK_1-5_ Ectodomain Can Adopt Multiple Conformations

We did not observe electron density for the first two domains, D1-D2, in the electron density map. SDS-PAGE of dissolved crystals showed a protein band corresponding to the last three Ig domains (Figure S2). Sequence analysis of potential protease cleavage sites revealed an internal furin site RGKR at positions 235–238, located between D2 and D3, suggesting that the ectodomain of OTK_1-5_ was cleaved by furin protease during crystallization. To prevent cleavage, we introduced a mutation K237A in the furin cleavage site; however, this construct did not provide sufficiently well-ordered crystals for data collection. Attempts to crystallize the D1-D2 domains alone were unsuccessful.

**Figure 2:**
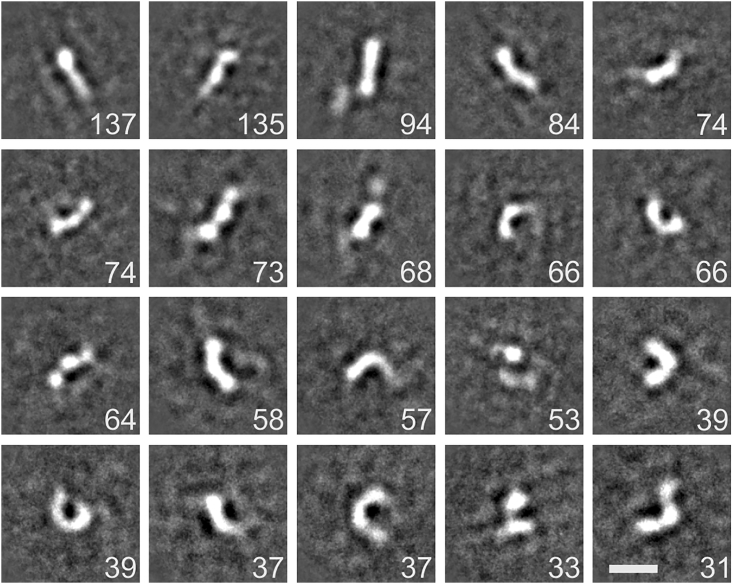
Conformational Flexibility of the OTK_1-5_ K237A Ectodomain Negative stain 2D class averages of the full-length OTK_1-5_ K237A ectodomain. Scale bar, 10 nm. The number of particles within each class is listed on the bottom right corner.

To examine the conformational flexibility of the full-length OTK_1-5_ ectodomain, we analyzed the structure of OTK_1-5_ K237A with single-particle negative-stain electron microscopy. In the micrographs, OTK_1-5_ K237A was monomeric. We calculated 25 2D class averages, which revealed a broad range of conformations (Figure 2). In the 2D class averages, the OTK_1-5_ K237A ectodomain showed a high flexibility with conformations ranging from fully extended, through the boomerang shape, to a C shape. This analysis supports our previous observation that OTK_3-5_ exhibits substantial interdomain flexibility and indicates that the full ectodomain can adopt multiple conformations.

### OTK Is a Monomeric Protein in Solution and on the Cell Surface

**Figure 3:**
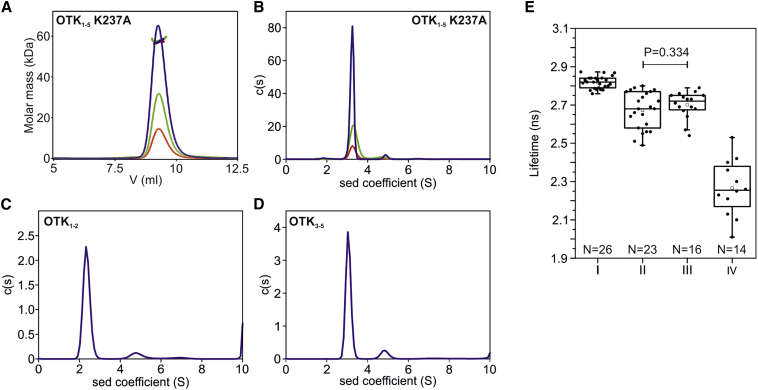
OTK Is a Monomer in Solution and on the Cell Surface (A) Size-exclusion chromatography with multi-angle light scattering indicates an experimental molar mass of 59 kDa for the OTK_1-5_ ectodomain, which is in agreement with the theoretical molar mass for a monomer (63 kDa). No peak shift toward higher molecular masses was observed at any of the initial protein concentrations of 2.0 mg/mL (blue), 1.0 mg/mL (green), and 0.5 mg/mL (red). (B) Sedimentation coefficient distribution of OTK_1-5_ K237A determined by sedimentation velocity analytical ultracentrifugation at a concentration of 5 mg/mL (blue), 3 mg/mL (green), and 1 mg/mL (red). The calculated molar mass of 66 kDa corresponds to the theoretical molar mass for a monomer. (C) Sedimentation coefficient distribution of OTK_1-2_ at a concentration of 1.3 mg/mL. The calculated molar mass of 30 kDa corresponds to the theoretical molar mass for a monomer (25 kDa). (D) Sedimentation coefficient distribution of OTK_3-5_ at a concentration of 1.8 mg/mL. The calculated molar mass of 44 kDa corresponds to the theoretical molar mass for a monomer (38 kDa). (E) FRET-FLIM in live COS-7 cells indicates that the cells co-expressing OTK-mClover and OTK-mRuby2 (II) show the similar lifetime to the cells expressing OTK-CD4-mClover and OTK-CD4-mRuby2 (III), in which a native transmembrane segment was replaced with a transmembrane segment of monomeric protein CD4. Both previous lifetimes are similar to the lifetime of donor alone, OTK-mClover (I). Cells expressing tandem mClover-mRuby2 were used as a positive control (IV). The box limits indicate the 25th and 75th percentiles, centered lines show the median, squares represent sample means, whiskers extend 1.5-fold the interquartile range from the 25th and 75th percentiles, the p value was calculated by one-way analysis of variance (ANOVA).

Analysis of crystal packing interactions revealed eight crystallographic contacts (Figure S3). The first one, with the largest total buried surface area of 2,404 Å^2^, results in a dimeric architecture between two molecules related by a crystallographic 2-fold symmetric axis, and is formed by the D4-D5 domains from each molecule. The next largest contact buries a total surface area of 2,113 Å^2^ and is primarily mediated by the D3-D4 domains from each molecule. The third largest contact with a total buried surface area of 1,604 Å^2^ is formed between the D3 and D4 domains. The other crystallographic contacts bury less than 900 Å^2^ of surface area. To analyze the oligomeric state of the OTK_1-5_ ectodomain released from the constraints of crystal packing, we performed multi-angle light scattering (MALS) and sedimentation velocity experiments. In solution, MALS indicated an experimental molecular mass of 59 kDa, which is in agreement with the theoretical mass for a monomer (Figure 3A). We did not observe a peak shift toward higher molecular masses at three different protein concentrations, indicating that there is probably no monomer-dimer equilibrium in solution. Sedimentation velocity experiments with higher protein concentrations also showed OTK_1-5_ to be a monomer; no propensity to multimerize was detected up to a concentration of 80 μM (Figure 3B). We then investigated whether cleavage of OTK_1-5_ ectodomain by furin results in a change of oligomeric state. Sedimentation velocity experiment revealed that, after cleavage, both purified OTK_1-2_ and OTK_3-5_ domains maintained their monomeric state; no propensity to multimerize was observed (Figures 3C and 3D).

A number of receptor tyrosine kinases have been reported to form dimers via their transmembrane segments (Li and Hristova, 2010). To investigate possible OTK oligomerization via the transmembrane segment, we constructed a mutant termed OTK-CD4, in which the native transmembrane segment was replaced with a transmembrane segment of the CD4 protein that does not contribute to oligomerization (Iliopoulou et al., 2018). Blue native PAGE followed by western blot analysis revealed no significant shift in electrophoretic mobility between OTK wild-type and OTK mutant (Figure S4A). Also, mild solubilization of cells expressing mClover-tagged OTK wild-type or OTK-CD4 mutant and subsequent fluorescent-detection size-exclusion chromatography (FSEC) showed no significant change in the peak position (Figure S4B). We then analyzed the potential for OTK oligomerization on live cell surfaces using FRET fluorescence lifetime imaging microscopy (FLIM). COS-7 cells were co-transfected with the FRET pairs OTK-mClover and OTK-mRuby2 or OTK-CD4-mClover and OTK-CD4-mRuby2 and the lifetime of the donor was measured. We did not observe significant shortening of the average lifetime for the cells expressing OTK wild-type relative to the cells expressing OTK-CD4, suggesting that OTK is present on the cell surface as a monomer (Figures 3E and S4C). These findings are consistent with our blue native PAGE and FSEC analyses.

### *Drosophila* OTK Interacts with Heparin

**Figure 4:**
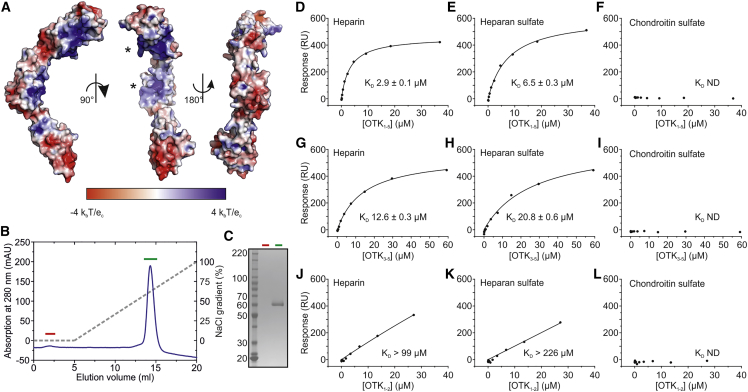
OTK_1-5_ K237A Ectodomain Binds Heparin And Heparan Sulfate (A) Surface representation of *Drosophila* OTK_3-5_ colored by electrostatic potential from −4k_b_T/e_c_ (red) to +4k_b_T/e_c_ (blue) shows two substantially basic regions at D3 and D4 (shown by asterisks). (B) Heparin affinity chromatography. OTK_1-5_ K237A was loaded onto a HiTrap Heparin column equilibrated with 15 mM HEPES (pH 7.4) and 50 mM NaCl, and eluted with a linear gradient to 1 M NaCl. OTK_1-5_ K237A was eluted at 550 mM NaCl concentration. (C) SDS-PAGE analysis of collected flow-through (red) and eluted peak fractions (green). (D–L) SPR equilibrium experiment. We tested binding between three analytes, OTK_1-5_ K237A (D–F), OTK_3-5_ (G–I), or OTK_1-2_ (J–L), and three ligands, heparin (D, G, and J), heparan sulfate (E, H, and K), or chondroitin sulfate (F, I, and L).

A close inspection of the electrostatic potential on the OTK_3-5_ surface revealed two substantial regions of basic charge on D3 and D4 (Figure 4A). The basic patch on D3 is formed by Lys285, Arg288, Arg292, Arg294, Lys295, Lys298, Arg301, Lys334, and Arg329, and the basic region on D4 is created by Lys386, Lys396, His398, Lys400, and Arg435. In the crystal structure, both basic patches interact with well-ordered sulfate ions from the crystallization solution (Figure S5A). Binding of sulfate ions and notable similarity to consensus sequences (Hileman et al., 1998) for heparin binding suggest that both patches represent potential glycosaminoglycan (GAG) binding sites. To examine whether OTK interacts with GAGs, we performed heparin affinity chromatography. We observed that OTK_1-5_ K237A bound to the heparin column and was eluted with a linear NaCl gradient at 550 mM NaCl concentration (Figures 4B and 4C). These results suggest that OTK can directly bind the GAG chains of proteoglycans. We made several attempts to produce and purify OTK constructs with point mutations in the putative GAG binding sites to prevent GAGs binding. In particular, we aimed to produce an OTK construct containing the mutations K237A, K396E, R435E, K386E, and H398D, or another construct containing the mutations K237A, K386A, and K396A. Although these mutants were expressed in our HEK293T-based expression system, the proteins were not secreted from the HEK239T cells and remained inside the cells. Therefore, it appears that the putative GAG binding sites are vital for OTK folding or secretion.

We further investigated the binding specificity of OTK toward GAGs using surface plasmon resonance (SPR) binding equilibrium experiments. We measured the affinity between OTK_1-5_ K237A and three GAGs, which were immobilized on the SPR chip surface. In particular, we tested heparin, heparan sulfate and chondroitin sulfate. In our SPR experiments, OTK_1-5_ K237A bound with greatest affinity to the most sulfated GAG, heparin, with an apparent K_D_ of 2.9 ± 0.1 μM, whereas binding to the second most sulfated GAG, heparan sulfate, was at least two times weaker (6.5 ± 0.3 μM). Conversely, we did not observe any measurable indication of binding between OTK_1-5_ K237A, and chondroitin sulfate when OTK_1-5_ K237A was present at concentrations up to 36.9 μM (Figures 4D–4F and S5). We further used OTK_1-2_ and OTK_3-5_ fragments to define the individual contributions of OTK domains to GAGs binding. OTK_3-5_ showed at least four times weaker binding to heparin and at least three times weaker binding to heparan sulfate compared with that of OTK_1-5_ K237A (Figures 4G and 4H). On the other hand, OTK_1-2_ bound heparin or heparan sulfate much more weakly, and we were not able to determine the K_D_s unambiguously (Figures 4J and 4K). Similarly to OTK_1-5_, both OTK_1-2_ and OTK_3-5_ showed no detectable binding to chondroitin sulfate (Figures 4I and 4L).

Taken together, our results reveal a correlation between the degree of GAG sulfation and the affinity to OTK with tighter binding occurring for higher GAG sulfation. This observation suggests that the electrostatic interactions between negatively charged sulfate groups and positively charged patches of OTK are the main drivers of GAG binding. Our binding experiments further indicate that both OTK_1-2_ and OTK_3-5_ contribute to GAG binding; however, the primary hotspot for GAG interaction appears to be located on OTK_3-5_.

### *Drosophila* OTK Interacts with Sema1a but Fails to Bind PlexA

**Figure 5:**
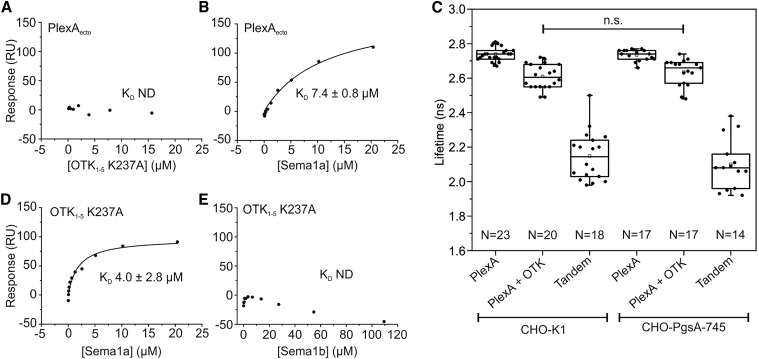
OTK ectodomain interacts with Sema1a but fails to bind PlexA (A) SPR equilibrium experiment indicates no interaction between the OTK_1-5_ K237A ectodomain and the PlexA_ecto_ ectodomain. (B) SPR equilibrium experiment with the ectodomain of Sema1a shows that immobilized PlexA is functional. (C) FRET-FLIM in live CHO-K1 cells, which express a number of proteoglycans on the cell surface, and CHO-PgsA-475 cells, which do not produce GAGs. FRET-FLIM indicates that the CHO-K1 cells co-expressing PlexA-mClover and OTK-mRuby2 show the similar average lifetime to the CHO-PgsA-475 cells expressing PlexA-mClover and OTK-mRuby2. The absence of significant differences in the average lifetimes suggests that GAGs do not mediate the interaction between PlexA and OTK on the cell surface. Furthermore, both lifetimes are similar to the lifetime of donor alone, PlexA-mClover. Cells expressing tandem mClover-mRuby2 were used as a positive control. The box limits indicate the 25th and 75th percentiles, centered lines show the median, squares represent sample means, whiskers extend 1.5-fold the interquartile range from the 25th and 75th percentiles, the p value was calculated by one-way ANOVA. (D) SPR equilibrium experiment indicates direct binding between OTK_1-5_ K237A and Sema1a. (E) SPR equilibrium experiment shows no binding between OTK_1-5_ K237A and Sema1b.

Previous studies demonstrated that OTK interacts biochemically and genetically with *Drosophila* PlexA (Winberg et al., 2001). To examine this interaction, we performed SPR binding equilibrium experiments. First, we probed binding between the OTK_1-5_ K237A ectodomain and the first four domains of PlexA_1-4_. For PlexA_1-4_ coupled to the SPR chip we did not observe any measurable indication of binding when OTK_1-5_ K237A was present at concentrations up to 125 μM (Figures S6A and S6B). We then used the full-length ectodomain of PlexA containing all ten domains instead of the first four domains. Again, we were not able to detect any measurable indication of binding between the OTK and PlexA ectodomains when OTK_1-5_ K237A was present at concentrations up to 125 μM (Figures 5A and S6C). We further examined the selective binding properties for the individual first two and last three OTK domains. However, in our SPR experiments, we again did not detect any measurable binding to PlexA neither for OTK_1-2_ nor OTK_3-5_ (Figures S6D–S6G). To demonstrate that the immobilized PlexA is functional, we performed an SPR experiment with the ectodomain of Sema1a, which has been previously reported to bind PlexA (Winberg et al., 1998). In our SPR experiment, Sema1a bound PlexA_ecto_ with an apparent K_D_ of 7.4 ± 0.8 μM (Figures 5B and S6H). This K_D_ is weaker than the previously reported affinity observed for the first four domains of PlexA_1-4_ (Rozbesky et al., 2019). The weaker binding affinity of PlexA_ecto_ may be caused by steric effects, for example an inhibitory intermolecular head-to-stalk interaction, similar to that previously shown for the ectodomains of mouse class A plexins (Kong et al., 2016). The lack of binding between OTK and PlexA in the SPR experiments indicated that an additional molecule might be required to mediate the previously reported OTK-PlexA interaction. *Drosophila* PlexA has been shown to bind heparin (Cho et al., 2012) and here we have demonstrated heparin binding for OTK. Therefore, we hypothesized that binding of PlexA and OTK might be indirect and mediated by GAGs. To examine this hypothesis, we performed FRET-FLIM measurements on the cell surface of CHO cells. CHO-K1 cells, which express a number of proteoglycans on the cell surface, were transiently co-transfected with PlexA-mClover and OTK-mRuby2. As a control, we used the CHO-PgsA-745 cell line, which is derived from the CHO-K1 cell line, but has a defect in xylosyltransferase, the first sugar-transfer enzyme in GAG synthesis, and thus does not produce GAGs. In the FRET-FLIM experiments, we observed an average lifetime of 2.61 ± 0.07 ns for CHO-K1 cells expressing PlexA-mClover and OTK-mRuby2, and this lifetime was similar to that observed for CHO-PgsA-745 expressing PlexA-mClover and OTK-mRuby2 (2.63 ± 0.08). Moreover, these average lifetimes are similar to those observed for cells expressing donor only (PlexA-mClover) indicating no or very low FRET (Figure 5C). The absence of significant differences in the average lifetimes indicates that GAGs do not mediate the interaction between PlexA and OTK on the cell surface. In light of these negative results, we set out to assess whether previously reported ligands of PlexA, Sema1a and Sema1b, interact with OTK. Unexpectedly, our SPR experiments revealed that the Sema1a ectodomain directly bound to OTK K237A with an apparent K_D_ of 4.0 ± 2.8 μM (Figures 5D and S6I). No binding was detected for the Sema1b ectodomain up to a concentration of 109 μM (Figures 5E and S6J) indicating that the interaction is specific for Sema1a. Taken together, our results suggest that instead of interacting with PlexA, the OTK ectodomain directly and specifically interacts with Sema1a.

## Discussion

Our data reveal several key properties of *Drosophila* OTK. First, we demonstrated that OTK is able to explore a large conformational space. The overall bending flexibility is primarily driven by mobility at the interdomain interfaces that is caused by the lack of strong interactions between the individual domains. This conformational plasticity may contribute to OTK function, as previously reported for several proteins containing Ig domains. For example, the interdomain flexibility of Ig domains plays a critical role in controlling filamin-ligand interactions (Lad et al., 2007, Seppala et al., 2015). Also, recent structural studies on Robo ectodomains suggest that flexibility of their Ig domains enable conformational changes between an autoinhibited and open, active conformation (Aleksandrova et al., 2018, Barak et al., 2019).

We further found that OTK can be cleaved by furin protease, which plays essential roles in embryogenesis, homeostasis, and disease (Thomas, 2002). In OTK, the furin cleavage site is located within the D2-D3 linker. Notably, the OTK2 ectodomain comprises only three Ig-like domains, corresponding to the D3-D5 domains of OTK. Thus, after furin cleavage, both OTK and OTK2 share the same domain organization in the ectodomain with a sequence identity of more than 61%. OTK has previously been reported to act as a neural cell adhesion molecule (Pulido et al., 1992). The predominant boomerang shape conformation we observed by electron microscopy for the full OTK ectodomain, and also in the OTK_3-5_ crystal structure, resembles that found for the first three Ig-like domains of the neural cell adhesion molecule (Soroka et al., 2003). Furthermore, a similar crescent shape has been shown to be essential for classical cadherin-mediated adhesion (Boggon et al., 2002, Harrison et al., 2011). Structurally, D4 and D5 are most similar to the fifth Ig-like domain (D5) in human Robo1, which has been implicated in an inhibitory Robo1-Robo2 *trans* interaction between opposing cells (Barak et al., 2019). In the crystal, OTK_3-5_ molecules showed extensive interactions with symmetry-related molecules; however, neither for OTK_1-5_ nor OTK_3-5_ were we able to detect any propensity to dimerize or multimerize in solution. Nevertheless, the lack of self-association observed for OTK_1-5_ in our experiments do not preclude it functioning in cell adhesion as the binding affinities determined for adhesion molecules are usually extremely weak (80–720 μM for E-cadherins) (Shapiro and Weis, 2009). Our FRET-FLIM measurement and native PAGE and FSEC analyses revealed that OTK is likely a monomer on the cell surface. These findings argue against previous studies, based on co-immunoprecipitation, showing that OTK can form homo-oligomers on the cell surface and that the interaction between OTK molecules is mediated by their transmembrane segment (Linnemannstons et al., 2014).

We have further discovered that GAGs are ligands for *Drosophila* OTK. In particular, we found that OTK binds heparin and heparan sulfate with apparent K_D_ values in the micromolar range, while no binding was observed for chondroitin sulfate. Binding between OTK and GAGs is likely mediated by electrostatic interactions and depends on the degree of sulfation. Putative GAG binding sites comprise two basic regions located on the D3 and D4 domains. The key positively charged residues that form the basic patches in OTK are conserved in OTK2, suggesting that OTK2 is also a GAG binding protein. An increasing body of evidence in recent years points to the importance of heparan and chondroitin sulfate proteoglycans in neural development. A number of axon guidance molecules have been shown to bind heparan or chondroitin sulfate proteoglycans, including netrin1, Slit2, ephrinA1, ephrinA5, Sema5B, Sema5A, and Sema3A (Maeda, 2015). Intriguingly, heparan or chondroitin sulfate proteoglycans have been reported as key mediators that can switch Sema5A from having an attractive effect to a repulsive effect (Kantor et al., 2004). In *Drosophila*, loss of function of the heparan sulfate proteoglycan perlecan resulted in motor axon defects resembling those of loss-of-function mutations in either Sema1a or PlexA. These data suggest that perlecan is an essential component of embryonic Sema1a-PlexA-mediated motor axon guidance *in vivo* (Cho et al., 2012). Molecular mechanisms governing the interplay between axon guidance cues and GAGs are poorly understood. One possible explanation might be receptor oligomerization and clustering, which has been shown for receptor protein tyrosine phosphatase σ, a receptor for both chondroitin sulfate and heparan sulfate proteoglycans (Coles et al., 2011). GAGs have also emerged as key players in the regulation of Wnt signaling (Ai et al., 2003, Dejima et al., 2014, Munoz et al., 2006, Saied-Santiago et al., 2017). Because OTK has been shown to bind a number of Wnts (Linnemannstons et al., 2014, Peradziryi et al., 2011), all three components, OTK, GAGs, and Wnts, might function in concert.

OTK has been reported to bind numerous structurally divergent ligands, including the cell surface receptor PlexA. Although binding between OTK and PlexA has been shown by genetic analysis and co-immunoprecipitation (Winberg et al., 2001), we failed to detect direct binding *in vitro* in our SPR binding experiments. Our FRET-FLIM experiments on the surface of live cells provide no evidence for binding between PlexA and OTK, we see neither direct, nor indirect GAG-mediated interactions. Unexpectedly, we found that instead of PlexA, OTK interacts with the Sema1a ectodomain. This finding is consistent with previously reported genetic analysis showing that loss of OTK produced phenotypes resembling those of loss-of-function mutations of either Sema1a or PlexA. Therefore, it appears that the PlexA-OTK interaction is indirect and mediated by Sema1a. However, further work will be necessary to tease out how the interplay between OTK and its interaction partners modulate cell signaling.

## STAR★Methods

### Key Resources Table

**Table:** 

REAGENT or RESOURCE	SOURCE	IDENTIFIER
**Chemicals, Peptides, and Recombinant Proteins**		

Dulbecco’s Modified Eagle Medium, high glucose	Sigma-Aldrich	Cat# D5796
Fetal Bovine Serum	Gibco	Cat# 12676029
Polyethylenimine, branched	Sigma-Aldrich	Cat# 408727
Pyrobest DNA Polymerase	Takara	Cat# R005A
D-biotin	Sigma-Aldrich	Cat# B4639
dodecyl β-D-maltoside	Antrace	Cat# D310
cholesteryl hemisuccinate	Antrace	Cat# CH210
Heparin sodium salt	Toronto Research Chemicals	Cat# H245800
Heparan sulfate	Toronto Research Chemicals	Cat# H245780
Chondroitin sulfate sodium salt	Toronto Research Chemicals	Cat# C432735
EZ-Link Biotin-LC-Hydrazide	ThermoFisher Scientific	Cat# 21340

**Deposited Data**		

Drosophila OTK, extracellular domains 3-5	This paper	PDB: 6S9F
MuSK	Stiegler et al., 2006	PDB: 2IEP
Robo1	Morlot et al., 2007	PDB: 2V9T
SYG-1	Ozkan et al., 2014	PDB: 4OF6

**Experimental Models: Cell Lines**		

HEK293T	ATCC	CRL-3216
HEK293S-GnTI^−^	ATCC	CRL-3022
COS-7	ATCC	CRL-1651
CHO-K1	ATCC	CCL-61
CHO-pgsa-745	ATCC	CRL-2242

**Oligonucleotides**		

Forward primer for OTK_1-5_: ATCCCGGGAGCTCATCGCG	This paper	N/A
Reverse primer for OTK_1-5_: AGGGTACCTCGGGTGACC	This paper	N/A
Reverse primer for OTK- mClover/mRuby2:TAGG TACCGCGATAGCG ACACCAC	This paper	N/A
Reverse primer for OTK-CD4: ATGGTACCGCGATAGCG ACAGAAGAAGATGCCTA GCCCAATGAAAAGCAG GAGGCCGGCGACGCC CCCCAGCACAATCAG GGCCATAGCTCGGGT GACCAGGAAGCC	This paper	N/A
Forward mutagenic primer for OTK K237A:CAGACCTTCCTGT GCCGCGGTGCGCGCGGTGG AGCTGCTGGACTAG	This paper	N/A
Reverse mutagenic primer for OTK K237A:CTAGTCCAGCA GCTCCACCGCGCGCACCGC GGCACAGGAAGGTCTG	This paper	N/A

**Recombinant DNA**		

Vector: pHLsec	Aricescu et al., 2006	N/A
Vector: pHL-Avitag3	Aricescu et al., 2006	N/A
Plasmid: pDisplay-BirA-ER	Howarth et al., 2008	N/A
Drosophila OTK cDNA	Provided by Prof Alex Kolodkin (Johns Hopkins University School of Medicine)	N/A
Synthetic gene OTK_1-5_-3C (GeneArt, Invitrogen)	This paper	N/A

**Software and Algorithms**		

Xia2	Winter, 2010	https://xia2.github.io
AIMLESS	Evans, 2006, Evans, 2011	http://www.ccp4.ac.uk/dist/html/aimless.html
DIALS	Winter et al., 2018	https://dials.github.io
PHASER	Mccoy et al., 2007	http://www.ccp4.ac.uk/html/phaser.html
COOT	Emsley and Cowtan, 2004	http://www2.mrc-lmb.cam.ac.uk/personal/pemsley/coot/
PHENIX	Afonine et al., 2012	https://www.phenix-online.org/
PDBePISA	Krissinel and Henrick, 2007	https://www.ebi.ac.uk/pdbe/pisa/
PDBeFold	Krissinel and Henrick, 2004	https://www.ebi.ac.uk/msd-srv/ssm/
APBS	Baker et al., 2001	http://www.poissonboltzmann.org/
MolProbity	Davis et al., 2007	molprobity.biochem.duke.edu
PyMOL	Schrodinger, LLC	https://www.pymol.org/
Corel Draw	Corel Corporation	https://www.coreldraw.com
ASTRA software	Wyatt Technology	https://www.wyatt.com/
SEDFIT	Schuck, 2000	http://www.analyticalultracentrifugation.com/default.htm
Eman2	Tang et al., 2007	https://blake.bcm.edu/emanwiki/EMAN2
Modeller	Fiser et al., 2000, Sali and Blundell, 1993	https://salilab.org/modeller/
Gromacs	Hess et al., 2008	http://www.gromacs.org/
AMBER99SB-ILDNP^∗^ force field	Best and Hummer, 2009, Lindorff-Larsen et al., 2010	http://www.gromacs.org/Downloads/User_contributions/Force_fields
P-LINCS algorithm	Hess, 2008	http://manual.gromacs.org/documentation/2019-rc1/reference-manual/algorithms/constraint-algorithms.html#the-lincs-algorithm
Symphotime	PicoQuant	https://www.picoquant.com/
Biacore T200 Evaluation software	GE Healthcare	https://www.biacore.com/
OriginPro v9.1	OriginLab	https://www.originlab.com/origin

**Other**		

HisTrap FF	GE Healthcare	Cat# 17-5255-01
HiTrap Heparin HP column	GE Healthcare	Cat# 17040601
Superdex 16/60 200 PG HiLoad	GE Healthcare	Cat# 28989335
Superdex 10/300 200 GL Increase	GE Healthcare	Cat# 28990944
Superose 6 3.2/300 Increase	GE Healthcare	Cat# 29091598
QuixStand	GE Healthcare	Cat# 56-4107-78
Biacore T200	GE Healthcare	Cat# 28975001
Sensor Chip SA	GE Healthcare	Cat# 29104992
DAWN HELEOS II	Wyatt Technology	N/A
Optilab rEX	Wyatt Technology	N/A
Optima XL-I analytical ultracentrifuge	Beckman Coulter	N/A
Leica SP8-X-SMD confocal microscope	Leica Microsystems	N/A
PicoHarp 300 module	PicoQuant	N/A

### Lead Contact and Materials Availability

Further information and requests for resources and reagents should be directed to and will be fulfilled by the Lead Contact who is Prof Yvonne Jones (yvonne@strubi.ox.ac.uk). All unique/stable reagents generated in this study are available from the Lead Contact with a completed Materials Transfer Agreement.

### Experimental Model and Subject Details

HEK293T, HEK293S-GnTI^−^, COS-7, CHO-K1 and CHO-pgsa-745 cells were cultured in DMEM supplemented with 10% of fetal bovine serum at 37°C and 5% CO_2_.

### Method Details

#### Protein Production

A construct encoding *Drosophila melanogaster* OTK_1-5_ (residues 23-580, UniProt: Q6AWJ9) was cloned into the pHLsec vector (Aricescu et al., 2006) in-frame with a C-terminal hexahistidine (His6) tag. For crystallization, OTK_1-5_ was produced by transient transfection in HEK293S-GnTI^−^ (ATCC CRL-3022) cells at 37°C. For all other experiments, OTK_1-5_ was produced in HEK293T (ATCC CRL-3216) cells at 37°C. The conditioned medium was collected 5-7 days post-transfection and proteins were purified from buffer-exchanged media by immobilized metal-affinity (HisTrap FF column, GE Healthcare) and size-exclusion chromatography (Superdex 200 16/60 column, GE Healthcare) in 15 mM HEPES (pH 7.4) and 150 mM NaCl. For OTK_1-2_ and OTK_3-5_ fragments production, a furin cleavage site (RGKR at position 235-238) located between D2 and D3 domains was replaced with HRV 3C cleavage site and this construct, OTK_1-5_-3C (commercially synthesized by GeneArt, Invitrogen), was produced and purified as wild-type OTK_1-5_. Purified OTK_1-5_-3C was cleaved with HRV-3C protease (1:100 w/w) for 16 hours at 6°C and OTK_1-2_ and OTK_3-5_ were separated by immobilized metal-affinity (HisTrap FF column, GE Healthcare) and size-exclusion chromatography (Superdex 200 16/60 column, GE Healthcare) in 15 mM HEPES (pH 7.4) and 150 mM NaCl. Site-directed mutagenesis of OTK_1-5_ was carried out by overlap-extension PCR, and the resulting PCR products were cloned into the pHLsec vector as described above.

#### Protein Crystallization, Data Collection and Structure Determination

Crystallization trials were set up using a Cartesian Technologies pipetting robot and consisted of 100 nl protein solution and 100 nl reservoir solution (Walter et al., 2005). All crystals were grown at 20°C in sitting drops using vapour diffusion. Prior to crystallization, purified OTK_1-5_ was concentrated to 7.0 mg/ml, supplemented with NDSB256 to a final concentration of 150 mM, and treated with endoglycosidase F1 (1:100 w/w) for 1 hour at 37°C. OTK_1-5_ crystallized in 0.05 M HEPES (pH 7.0), 0.01 M magnesium chloride, 150 mM NDSB256 and 1.6 M ammonium sulfate. Crystals were cryoprotected by soaking in reservoir solution supplemented with 25% (v/v) glycerol and then flash-cooled in liquid nitrogen.

Diffraction data were collected at 100K at the Diamond Light Source beamline I03 and indexed, integrated and scaled using the automated XIA2 (Winter, 2010), AIMLESS (Evans, 2006, Evans, 2011) and DIALS (Winter et al., 2018). The structure of OTK_3-5_ was initially solved by molecular replacement in PHASER (Mccoy et al., 2007) using the structures of MuSK (Stiegler et al., 2006) (PDB: 2IEP), Robo1 (Morlot et al., 2007) (PDB: 2V9T) and SYG-1 (Ozkan et al., 2014) (PDB: 4OF6), as search models. The partial model was completed by several cycles of manual rebuilding in COOT (Emsley and Cowtan, 2004) and refinement in PHENIX (Afonine et al., 2012). We were not able to model the βC’-βD loop (residues 309-328) in D3 completely because of fragmentary electron density. The final model was validated with MolProbity (Davis et al., 2007). Data collection and refinement statistics are given in Table 1. Structural alignment was performed using PDBeFold (Krissinel and Henrick, 2004), buried surface areas of protein–protein interactions were calculated with PDBePISA (Krissinel and Henrick, 2007), and electrostatics potentials were generated with APBS (Baker et al., 2001). Figures were produced with PyMOL (Schrodinger, LLC) and Corel Draw (Corel Corporation).

#### Size-Exclusion Chromatography with Multi-Angle Light Scattering (SEC-MALS)

Proteins were injected onto the Superdex 200 Increase 10/300 column (GE Healthcare) at a flow rate of 0.5 ml/min in 15 mM HEPES (pH 7.4) and 150 mM NaCl. The SEC column was coupled with a static light-scattering (DAWN HELEOS II, Wyatt Technology), differential refractive index (Optilab rEX, Wyatt Technology) and Agilent 1200 UV (Agilent Technologies) detectors. The molecular mass of glycoproteins containing N-linked oligomannose-type sugars was determined using an adapted RI increment value (dn/dc standard value, 0.185 ml/g). Data were analysed using the ASTRA software (Wyatt Technology).

#### Analytical Ultracentrifugation

Sedimentation velocity experiments were performed using an Optima XL-I analytical ultracentrifuge (Beckman) operated at 20°C. Samples of OTK in 15 mM HEPES (pH 7.4) and 150 mM NaCl were centrifuged in double sector 12 mm centerpieces in an An-60 Ti rotor (Beckman) at 40000 rpm. Protein sedimentation was monitored by an absorption optical system and Rayleigh interference system. Data were analysed using SEDFIT (Schuck, 2000). A value of 0.73 mL/g was used for the partial specific volumes. A buffer density value of 1.00558 g/cm^3^ and buffer viscosity value of 0.01028 Poise was calculated using the Sednterp online application.

#### Single Particle Negative Stain Electron Microscopy

Freshly purified OTK_1-5_ K237A (8 μg/ml) in 15 mM HEPES (pH 7.4) and 150 mM NaCl was stained with 0.75% uranyl formate using the conventional negative staining protocol (Booth et al., 2011). Images were recorded using a Tecnai T12 transmission electron microscope operated at 120 kV on a 4000×4000 high-sensitivity FEI Eagle at a magnification of 67,000, which corresponds to 1.68 Å/pixel sampling of the specimen. A defocus value of about -1.5 μm was used. Particles were manually selected and processed using the Eman2 (Tang et al., 2007) software.

#### Molecular Dynamics Simulations

Molecular dynamics simulations of OTK_3-5_ were performed in Gromacs (Hess et al., 2008) using the AMBER99SB-ILDNP^∗^ force field (Best and Hummer, 2009, Lindorff-Larsen et al., 2010). The missing residues in OTK_3-5_ (residues 309-328) were modelled in Modeller(Fiser et al., 2000, Sali and Blundell, 1993). Before the simulation, the protein was immersed in a box of SPC/E water, with a minimum distance of 1.0 nm from the box edge. A total of 150 mM NaCl was added using genion. Long-range electrostatics were treated with the particle-mesh Ewald summation (Essmann et al., 1995), and bond lengths were constrained using the P-LINCS algorithm (Hess, 2008). The integration time step was 5 fs. The v-rescale thermostat and the Parrinello–Rahman barostat were used to maintain a temperature of 300 K and a pressure of 1 atm. Simulations were carried out in triplicates of 100 ns each. The system was energy minimized using 1000 steps of steepest descent and equilibrated for 200 ps with restrained protein heavy atoms. Snapshots were extracted every 500 ps from each trajectory.

#### Fluorescence Resonance Energy Transfer – Fluorescence Lifetime Imaging Microscopy (FRET-FLIM) in Live Cells

A construct encoding Drosophila OTK (residues 23-607) encompassing the ectodomain, a transmembrane segment and a short cytoplasmic linker was cloned into the pHLsec vector in-frame with a C-terminal fluorescent protein, mClover or mRuby2. A monomeric OTK, which we used as a control, was prepared by substitution of a native transmembrane segment with a transmembrane segment of human CD4 protein (UniProt: P01730).

COS-7 cells were cultured in a phenol red free DMEM supplemented with 10% of fetal bovine serum at 37°C on glass-bottom 35 mm Petri dishes (Mattek). Before imaging, COS-7 cells were transiently transfected with the FRET pairs OTK-mClover and OTK-mRuby2; a donor-only sample (OTK-mClover) or a fusion construct of mClover-mRuby2, which was used as a positive control.

FLIM experiments were performed two days post-transfection using a Time-Correlated Single Photon Counting (TCSPC) system operated by a PicoHarp 300 module (PicoQuant) attached to a Leica SP8-X-SMD confocal microscope (Leica Microsystems) with a 63×/1.40 numerical aperture oil immersion objective at 37°C. A 488 nm picosecond pulsed diode laser PDL 800-B (PicoQuant) tuned at 80 MHz was used to excite the donor and the emitted photons passing through the 500-550 nm emission filter were detected using an external hybrid detector in photon counting mode. At least 400 photon events per pixel were collected in all cases, and the lifetime analysis was carried out using a Symphotime (PicoQuant). The acquired fluorescent decays *i(t)* were fitted by mono- (Equation 1) or biexponential (Equation 2) model.

(Equation 1) $$i\left(t\right)=Ae^{-t/\tau_{1}}$$

(Equation 2) $$i\left(t\right)=A_{1}e^{-t/\tau_{1}}+A_{2}e^{-t/\tau_{2}}$$

In Equations 1 and 2 τ_1_ is the lifetime of the donor alone, τ_2_ is the lifetime of the donor in the presence of the acceptor, A, A_1_ and A_2_ are amplitudes. The average donor lifetime obtained from a mono-exponential fit from the cells expressing the donor only (PlexA-mClover) was fixed in the bi-exponential model to calculate the remaining two amplitudes and the second lifetime (Padilla-Parra et al., 2008, Padilla-Parra and Tramier, 2012). The amplitude weighted average lifetime of the donor (τ_av_) was calculated using the equation Equation 3:

(Equation 3) $$\tau_{av}=\sum_{i}A_{i}\tau_{i}/\sum_{i}A_{i}$$

#### Fluorescence-Detection Size-Exclusion Chromatography (FSEC)

HEK293T cells were transiently transfected in a six-well culture plate with mClover-tagged OTK wild-type or OTK-CD4 mutant as described in the previous section. Two days post-transfection, cells were washed with PBS and resuspended with 25 mM HEPES (pH 7.5), 300 mM NaCl, 1.0% mixture of n-dodecyl β-D-maltoside (DDM) and cholesteryl hemisuccinate (CHS) (5:1 w/w, Antrace), and a mixture of protease inhibitors (Roche). The cell suspension was spun down at 15000 g for 10 minutes at 4°C, and the resulting supernatant was loaded onto a Superose 6 Increase column 3.2/300 (GE Healthcare) at a flow rate of 0.08 ml/min in 15 mM HEPES (pH 7.4), 150 mM NaCl and 0.03% mixture of dodecyl maltoside and cholesteryl hemisuccinate (5:1, Antrace). Elution was monitored by a FSEC HPLC system (Shimadzu) at λ_Ex_/λ_Em_=515/528 nm.

#### Surface Plasmon Resonance Equilibrium Binding Experiments

For SPR binding experiments, constructs encoding PlexA_1-4_ (residues 28-730, UniProt: Q9V491), PlexA_ecto_ (residues 28-1272) or OTK_1-5_ (residues 23-580, UniProt: Q6AWJ9) were cloned into the pHL-Avitag3 vector (Aricescu et al., 2006) in frame with a biotin ligase recognition site followed by the C-terminal hexahistidine (His6) tag. *In vivo* biotinylation of OTK and PlexA constructs in HEK293T cells was performed by co-transfection with pDisplay-BirA-ER (Howarth et al., 2008) (pHLsec:pDisplay ratio was 3:1). To ensure near-complete biotinylation, a final concentration of 100 μM D-biotin was maintained in the DMEM medium. Two days post-transfection, conditioned medium was collected and dialysed against 15 mM HEPES (pH 7.2), 150 mM NaCl, 3 mM CaCl_2_ and 0.005% (v/v) Tween 20. Biotinylated OTK and PlexA constructs were immobilized onto SA Biocore sensor chips (GE Healthcare). Heparin, heparan sulfate and chondroitin sulfate (Toronto Research Chemicals) were biotinylated using EZ-Link Biotin-LC-Hydrazide (Thermo Fisher Scientific) according to the manufacturer’s instructions and immobilized onto SA Biocore sensor chips (GE Healthcare). SPR experiments were performed using a Biacore T200 instrument (GE Healthcare) in PBS and 0.05% (v/v) Tween 20 at 25°C. The signal from experimental flow cells was corrected by subtraction of the nearest blank injection and the reference signal from a blank flow cell. Surface regeneration was performed three times per run using a buffer containing 0.1 M Tris (pH 8.0), 0.5 M NaCl and 1% CHAPS. All data were analyzed with Biacore T200 evaluation software (GE Healthcare).

#### Heparin Affinity Chromatography

Heparin affinity chromatography was performed using a HiTrap Heparin HP column 1 ml (GE Healthcare). Purified OTK_1-5_ K237A (0.5 mg) was loaded at a flow rate of 1 ml/min onto the Heparin column equilibrated with 15 mM HEPES (pH 7.4) and 50 mM NaCl. After washing, OTK was eluted with a linear NaCl gradient to 1 M NaCl. Flow-through and peak fractions were analyzed by SDS-PAGE.

### Quantification and Statistical Analysis

Synchrotron data collection and refinement statistics are given in Table 1. Molecular dynamics simulations (Figure 1D) were carried out in triplicates of 100 ns each; the standard deviation was calculated in OriginPro v9.1. FRET-FLIM measurements (Figures 3E, 5C, and S4C) were performed in three independent experiments. The lifetime analysis was carried out using the Symphotime software (PicoQuant). The calculated lifetimes were plotted in OriginPro v9.1. The box limits indicate the 25th and 75th percentiles, centred lines show the median, squares represent sample means, whiskers extend 1.5-fold the interquartile range from the 25th and 75th percentiles, the p-value was calculated by one-way analysis of variance (ANOVA). SPR experiments were performed in duplicates. All SPR data were analyzed with Biacore T200 evaluation software (GE Healthcare). Data are presented as means ± standard deviations.

### Data and Code Availability

Structure factors and coordinates have been deposited in the Protein Data Bank with identification number PDB 6S9F.

## Supplemental Information

Document S1. Figures S1–S6

Document S2. Article plus Supplemental Information
