# Supplementary material for: Drosophila OTK Is a Glycosaminoglycan-Binding Protein with High Conformational Flexibility
Source: Structure. 2020 May 5;28(5):507–515.e5. doi: 10.1016/j.str.2020.02.008 (PMC7203548; doi:10.1016/j.str.2020.02.008)
Supplement: Document S1. Figures S1–S6 [file mmc1.pdf]

Structure, Volume *28*

Supplemental Information

Drosophila OTK Is a Glycosaminoglycan-Binding Protein with High Conformational Flexibility

Daniel Rozbesky, Jim Monistrol, Vitul Jain, James Hillier, Sergi Padilla-Parra, and E. Yvonne Jones

Figure S1

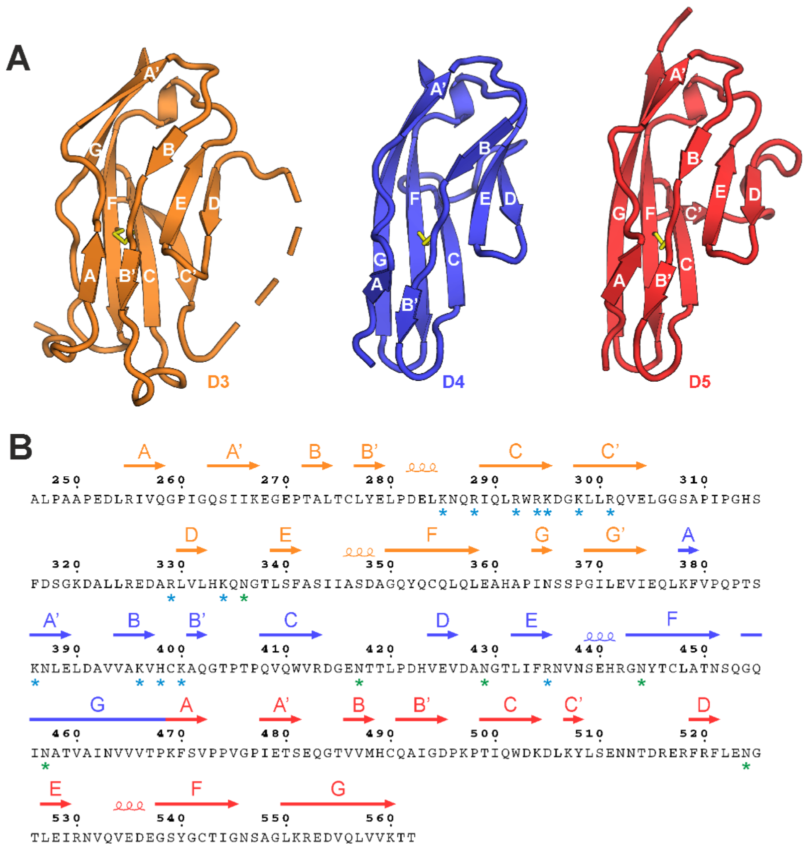

Figure S1 Structure of Drosophila OTK$_{3-5}$, Related to Figure 1

(A) Ribbon representation of single OTK domains; D3 domain is shown in orange, D4 domain in blue and D5 domain in red. Disulfide bonds are shown as yellow sticks. Secondary structure elements are labelled.

(B) Secondary structure elements are shown above the sequence of the OTK$_{3-5}$ domains. N-glycosylation sites are shown by green asterisks. The residues involved in heparin binding are shown by blue asterisks.

Figure S2

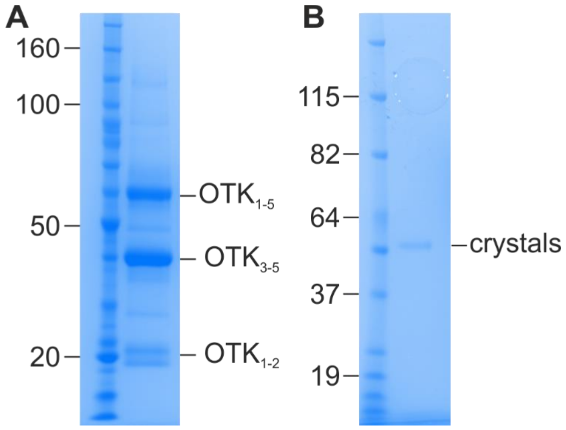

Figure S2 Cleavage of the $OTK_{1\text{-}5}$ ectodomain during crystallization, Related to Figure 1
(A) SDS-PAGE analysis of droplets from a crystallization plate revealed that the purified ectodomain of $OTK_{1\text{-}5}$ was cleaved probably by furin protease at position between D2 and D3 domains.
(B) SDS PAGE analysis of dissolved crystals showed a band that corresponds to $OTK_{3\text{-}5}$.

Figure S3

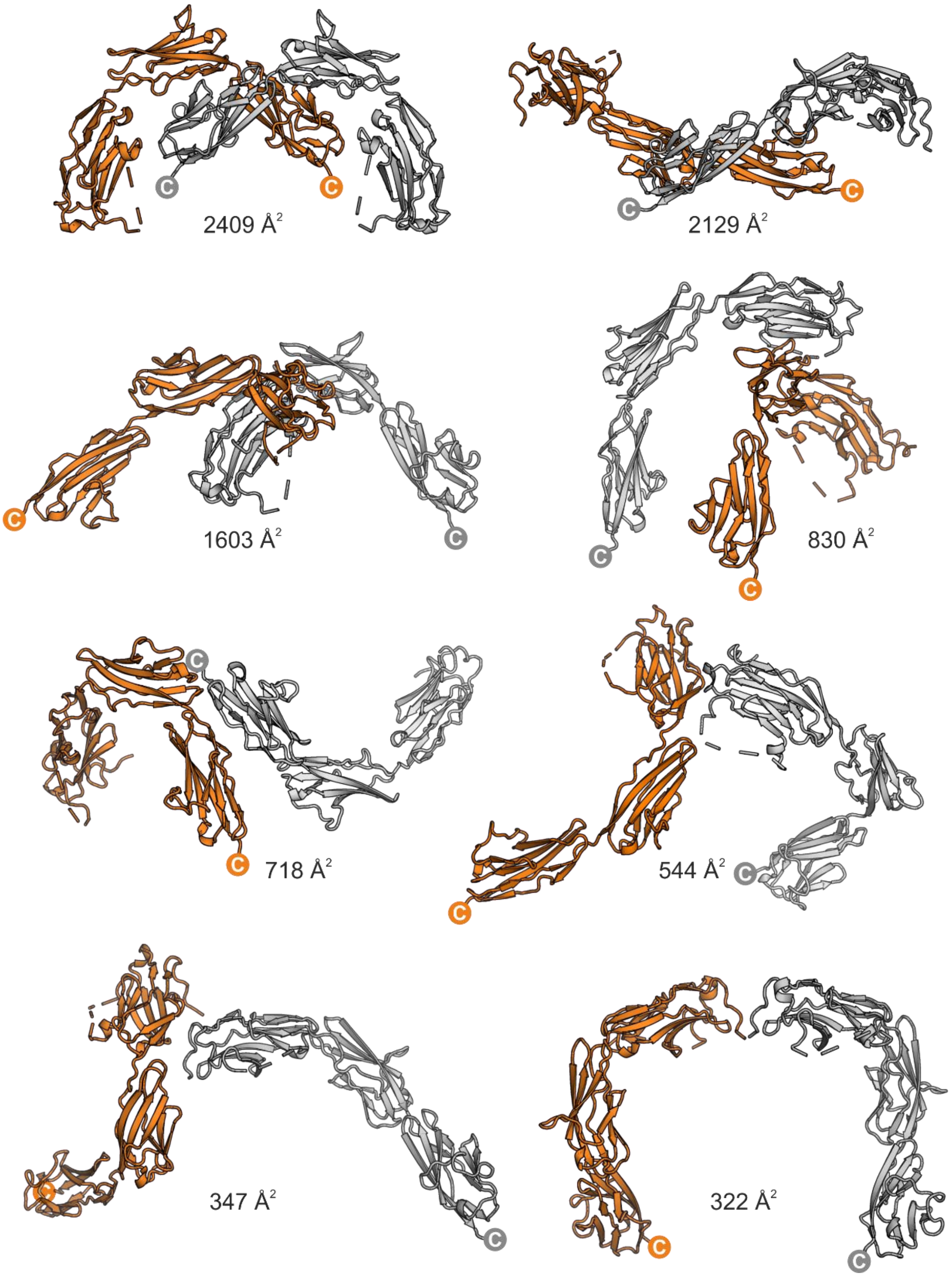

Figure S3 Crystallographic contacts between OTK$_{3-5}$ molecules, Related to Figure 1
Ribbon representation of crystallographic contacts between OTK$_{3-5}$ molecules. The total buried surface area of the interface was calculated by a PISA server and is shown below the ribbon representations.

Figure S4

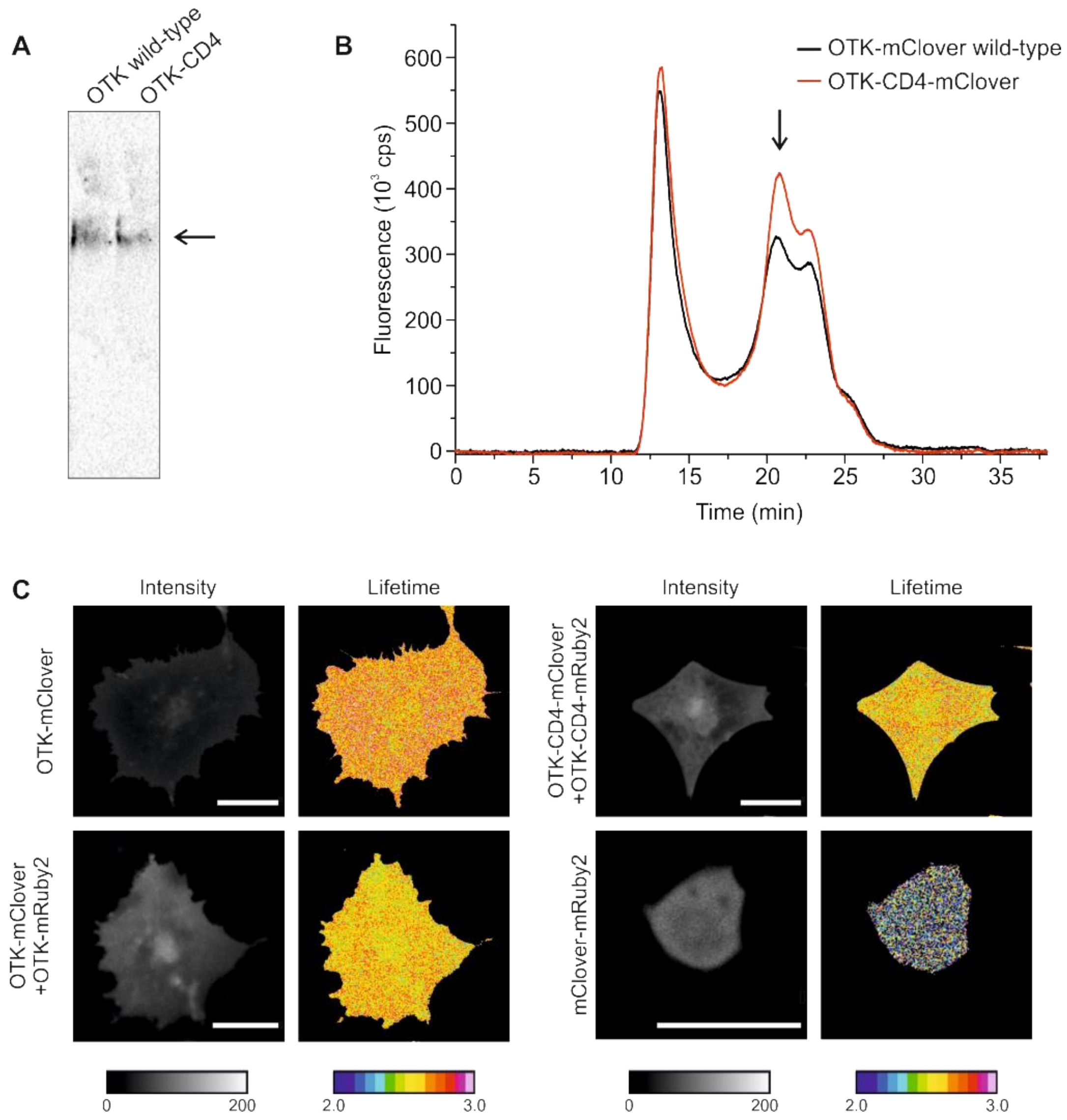

Figure S4 OTK is a monomer on the cell surface, Related to Figure 3

(A) HEK293T cells were transfected with OTK wild-type or OTK-CD4 mutant in which the native transmembrane segment was replaced with a transmembrane segment of CD4 protein. Two days post-transfection, cell lysates were used for Blue native PAGE followed by Western blot analysis with antiPentaHis antibody. No change in the electrophoretic mobility indicates that OTK is a monomer on the cell surface.

(B) Similarly, HEK293T cells were transfected with mClover tagged OTK wild-type or OTK-CD4 mutant, and two days post-transfection, cells were mildly solubilized with dodecyl maltoside and cholesteryl hemisuccinate. The resulting supernatants were analysed by FSEC. Consistent with Blue native PAGE, we did not observe a significant change in peak positions indicating that OTK wild-type is a monomer on the cell surface.

(C) FRET-FLIM analysis of OTK on the cell surface. Representative intensity and FLIM images of COS-7 cells transiently expressing FRET donor (OTK-mClover or OTK-CD4-mClover), FRET acceptor (OTK-mRuby2 or OTK-CD4-mRuby2) or tandem mClover-mRuby2 . The FLIM images are pseudocolored. Scale bar, 40 µm.

Figure S5

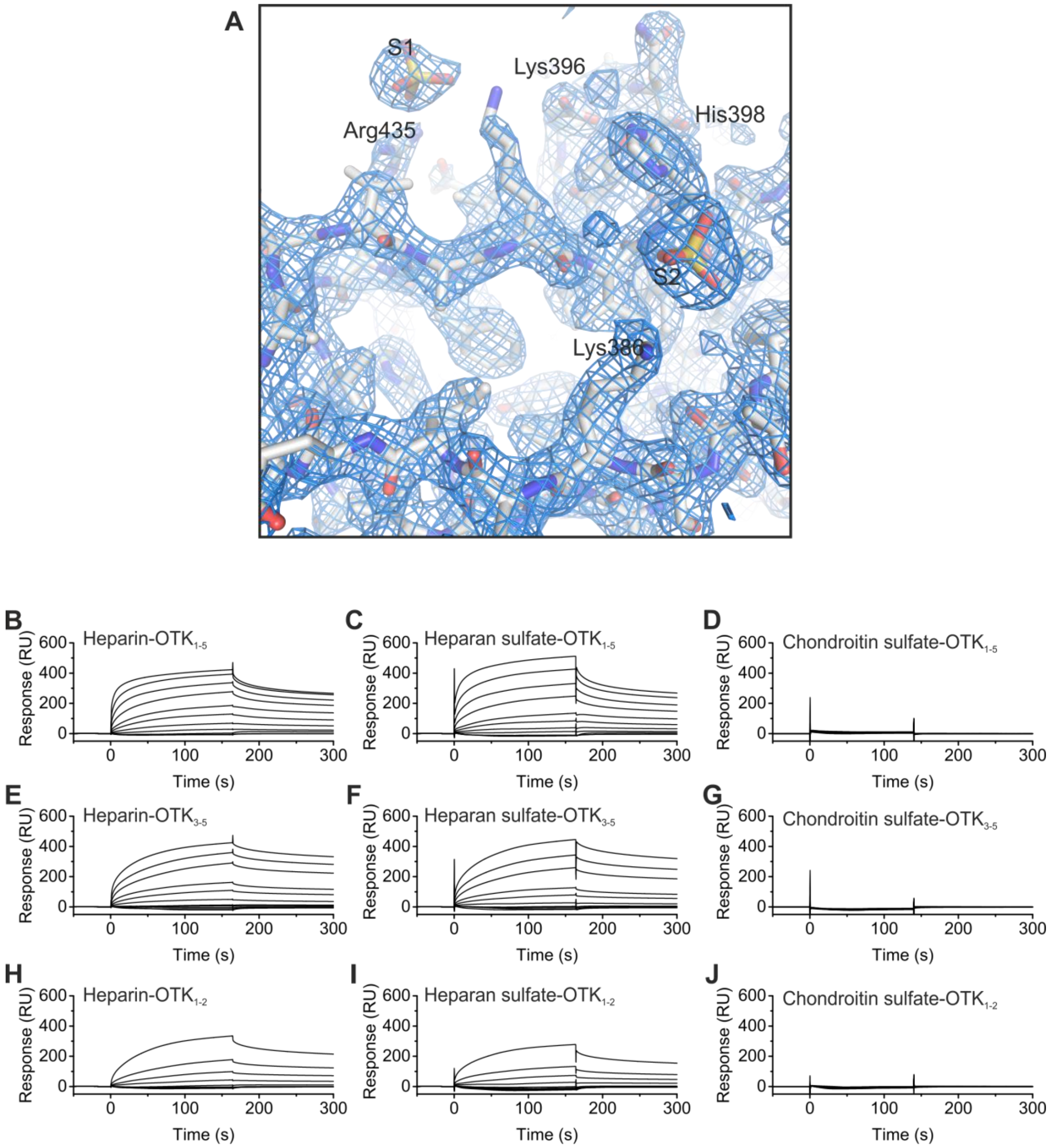

Figure S5 OTK binding to heparin and heparan sulfate, Related to Figure 4

(A) Close-up view showing binding of sulfate ions to the D4 domain of OTK_{3-5}. Two sulfate ions (S1 and S2) from the crystallization solutions are bound to the basic region of the D4 domain. The OTK_{3-5} structure is overlaid with the $2mF_o - DF_c$ map (calculated with Phenix for the final refined model) shown in blue at the contour level of 1.5 σ.

(B-J) Representative SPR sensograms showing OTK binding to heparin and heparan sulfate. We tested binding between three analytes, OTK_{1-5} K237A (B-D) or OTK_{3-5} (E-G) or OTK_{1-2} (H-J), and three ligands, heparin (B, E, H) or heparan sulfate (C, F, I) or chondroitin sulfate (D, G, J).

Figure S6

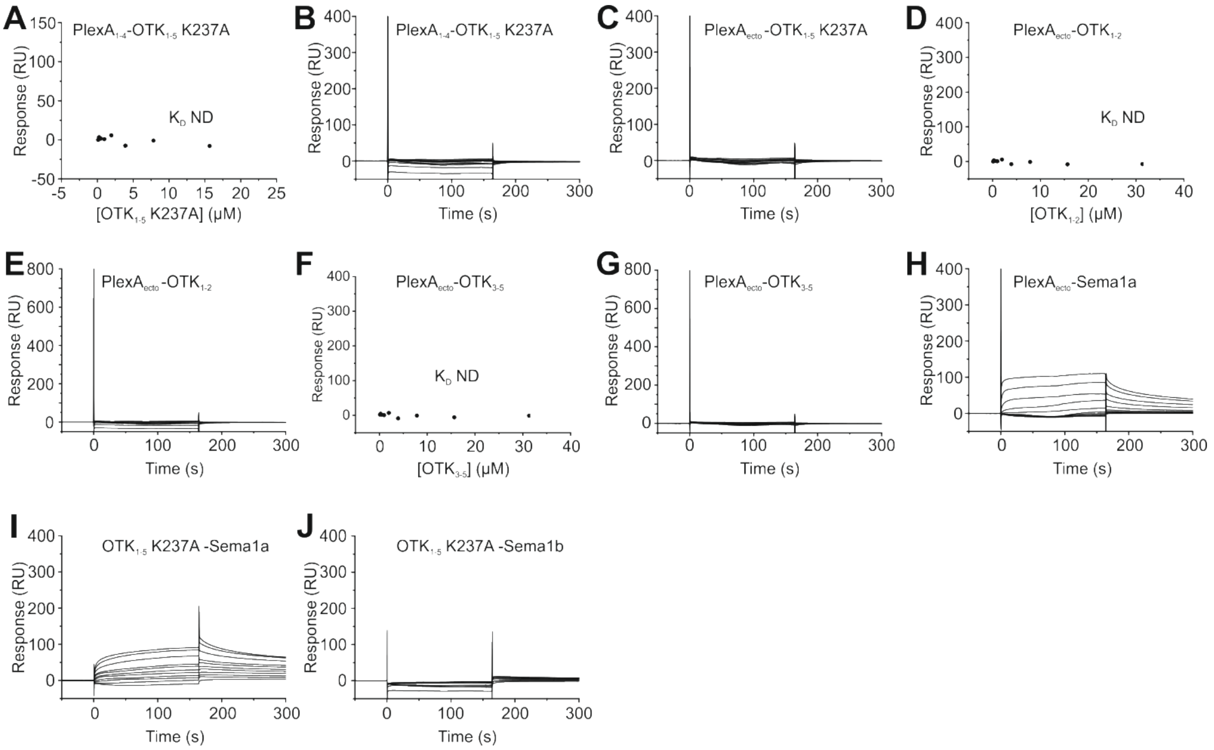

Figure S6 SPR experiments between OTK and PlexA, Sema1a or Sema1b, Related to Figure 5

(A) SPR equilibrium experiment indicates no interaction between the $OTK_{1\text{-}5}$ K237A ectodomain and first four domains of $PlexA_{1\text{-}4}$

(B) Representative SPR sensogram for the analysis shown in (A).

(C) Representative SPR sensogram for the $PlexA_{ecto}$ ectodomain and $OTK_{1\text{-}5}$ K237A.

(D-G) SPR equilibrium experiment indicates no interaction between $OTK_{1\text{-}2}$ (D-E) or $OTK_{3\text{-}5}$ (F-G) and $PlexA_{ecto}$.

(H) Representative SPR sensogram for the $PlexA_{ecto}$ ectodomain and Sema1a.

(I-J) Representative SPR sensogram for $OTK_{1\text{-}5}$ K237A and Sema1a (I) or Sema1b (J).
